# A Simple Micromilled Microfluidic Impedance Cytometer with Vertical Parallel Electrodes for Cell Viability Analysis

**DOI:** 10.3390/mi14020283

**Published:** 2023-01-22

**Authors:** Jason Eades, Julianne F. Audiffred, Micah Fincher, Jin-Woo Choi, Steven A. Soper, William Todd Monroe

**Affiliations:** 1 Department of Biological and Agricultural Engineering, Louisiana State University and Agricultural Center, Baton Rouge, LA 70803, USA; 2 Department of Electrical and Computer Engineering, Michigan Technological University, Houghton, MI 49931, USA; 3 Department of Chemistry, University of Kansas, Lawrence, KS 66044, USA; 4 Center of Biomodular Multiscale Systems for Precision Medicine, University of Kansas, Lawrence, KS 66044, USA

**Keywords:** single cell, microfluidic impedance cytometry, impedance spectroscopy, micromilling, vertical electrodes, sidewall electrodes

## Abstract

Microfluidic impedance cytometry has been demonstrated as an effective platform for single cell analysis, taking advantage of microfabricated features and dielectric cell sensing methods. In this study, we present a simple microfluidic device to improve the sensitivity, accuracy, and throughput of single suspension cell viability analysis using vertical sidewall electrodes fabricated by a widely accessible negative manufacturing method. A microchannel milled through a 75 µm platinum wire, which was embedded into poly-methyl-methacrylate (PMMA), created a pair of parallel vertical sidewall platinum electrodes. Jurkat cells were interrogated in a custom low-conductivity buffer (1.2 ± 0.04 mS/cm) to reduce current leakage and increase device sensitivity. Confirmed by live/dead staining and electron microscopy, a single optimum excitation frequency of 2 MHz was identified at which live and dead cells were discriminated based on the disruption in the cell membrane associated with cell death. At this frequency, live cells were found to exhibit changes in the impedance phase with no appreciable change in magnitude, while dead cells displayed the opposite behavior. Correlated with video microscopy, a computational algorithm was created that could identify cell detection events and determine cell viability status by application of a mathematical correlation method.

## 1. Introduction

Elucidation of the heterogeneities inherent to all populations of biological cells has a tremendous impact on our understanding of disease mechanisms and development of effective treatments [1,2]. As a result, considerable effort has been directed to the advancement of tools capable of measuring cellular biophysical properties on a single-cell basis at a sufficient throughput to gather population-level data. Many traditional methods for single-cell property measurement analyze one cell or a small population of cells in batch conditions and are therefore unsuitable for this purpose [3]. Flow cytometry offers a rapid, high-throughput method for single-cell analysis, but conventional flow technologies, such as fluorescence-activated cell sorting (FACS), require extensive sample processing for conjugation of fluorescent labels with cells of interest prior to cytometric detection. Such label-dependent strategies are necessarily labor-intensive and invasive to cells and can render samples unsuitable for many subsequent uses [4]. Additionally, while optical flow cytometry techniques have been proven effective in probing biochemical properties, conventional methods are not well suited to analysis of cellular biophysical properties. Alternatively, analysis of cells based on their dielectric properties is an inherently rapid, non-invasive, and label-free approach and provides the opportunity for direct correlation of measurements to relevant biophysical properties for electrical phenotyping.

The advent of microfluidic technology has provided a means to overcome many of the challenges associated with single cell analysis by reducing the cost and complexity of integrated tools while increasing sensitivity and throughput [5]. In 2001, Gawad et al. [6] demonstrated a microfabricated impedance flow cytometer, which combined the advantages of microfluidic technology with the basic functionality of standard electrical analysis methods for continuous flow-through single-cell dielectric property measurements. The mechanism employed by this device fundamentally involved passing cells through a microchannel flanked by a pair of electrodes, applying a voltage potential between the electrodes, and measuring the differential current change caused by the dielectric impedance of the cell and suspending buffer. By combining an understanding of the frequency dependence of the current response induced by biological cells with simplified electrical models of a cell, impedance measurements can be correlated to important biophysical characteristics including the size and shape of the cell, properties of its membrane, conditions of intracellular structures, etc. However, while many efforts have necessarily been directed to the advancement of novel device designs and informational multiparametric (e.g., multifrequency) analyses, the cost of the many tools required for the fabrication and operation of these devices may be prohibitive to some users. As a result, in addition to the development of greater analysis capabilities, implementation of widely accessible fabrication and measurement tools should be considered [7].

Accurate and sensitive application of impedance cytometry relies on the optimization of key features of the device design and operating conditions. There are two standard electrode configurations commonly utilized on microchips for impedance analysis of single cells: coplanar and top/bottom parallel geometries. Coplanar electrode geometries, in which electrodes are arranged on the same physical plane, are often used due to the relative simplicity of their fabrication. However, the electric field generated by these geometries is highly nonuniform, which leads to a significant positional dependence of measured impedance values on the height of the cell in the channel that cannot be easily resolved to generate accurate measurements. As a result, various methods have been devoted to the mitigation of this positional dependence including the use of flow focusing techniques [8,9,10], novel electrode configurations [9,11,12,13], and advanced signal processing methods [14]. The reader is directed to the recent review of methods used to account for or to reduce positional dependence by Daguerre et al [15]. However, in many cases, these alternative methods require complex fluid manipulation strategies, result in reduced sensitivity, and/or perform measurements using more than two electrodes, which increases the risk of coincidence events (in which impedance measurements are distorted by the presence of multiple cells within the detection region). Conversely, in parallel electrode geometries, the generated electric field is much more homogeneous and the dependence of measurements on cell position is significantly reduced throughout most of the detection region. Some commercially available cell analysis systems have been developed using top/bottom facing electrode configurations for analysis of pollen [16] and bacteria [17] as well as cell counting and viability testing [18]; however, others have noted that fabrication of electrodes in this orientation is complex and time-consuming, limiting its widespread adoption [19]. Despite the advantages of electric field homogeneity offered by parallel electrodes, coplanar geometries are often favored as standard methods for fabrication of devices with top/bottom parallel electrode geometries in a microfluidic chip are complex [20] and require advanced alignment capabilities. Vertical parallel sidewall electrode geometries represent an alternative facing electrode geometry that generate vertically homogeneous electric fields [21]. To date, the inclusion of vertical electrodes has not been widely adopted in the field of microfluidic impedance cytometry. However, advances in subtractive manufacturing techniques provide new opportunities for rapid, low-cost fabrication of microscale features with geometries that have otherwise been difficult to access.

Properties of the cell-carrying buffer should also be optimized to maximize the contribution of cells to measured impedance values and thereby maximize device sensitivity. Typically, cells are suspended in phosphate-buffered saline (PBS) for biologically relevant analyses. However, while this solution does effectively maintain physiologic osmolality and pH (thereby not affecting cell viability), its relatively high electrical conductivity can lead to a significant portion of the applied electric field bypassing cells. Constriction channels [22] and constraining sheath flows [8] have been implemented in attempts to mitigate current leakage and increase sensitivity. However, these techniques risk channel clogging and require additional instrumentation for flow control, respectively. Conversely, the use of buffers consisting of diluted PBS [17] and of a PBS-sucrose mixture [23] have been used for impedance flow cytometry, demonstrating the potential advantages of low-conductivity buffers for optimization of sensitivity.

The difficulty of achieving sufficient sensitivity for impedance flow cytometry is also reflected in the extensive efforts undertaken, as reviewed recently by Honrado et al. [7], to effectively process impedance signatures and accurately identify cell detection events. These events can be isolated from raw signals among noise and baseline drift by correlation to a mathematical function that corresponds to the expected profile. The electric field shape, and therefore the appropriate template function, is dictated by device design parameters including electrode configuration and dimensions, microchannel width and height, buffer properties, interrogation frequency, and cell biophysical properties and location [14,24,25]. As a result, analytical or numerical analysis tools should be applied to identify the expected impedance profile for a given device design and to evaluate the effects of positional dependence on that profile [15].

Microfluidic impedance cytometry has gained significant interest as a cell viability detection method that can be integrated directly with various other biological analysis tools [26,27]. Specifically, the application of microfluidic impedance cytometry for viability analysis of T lymphocyte cells has been tested previously by others [28,29], but each of these devices required the use of constriction channels or ancillary particle focusing or trapping tools that reduced their throughput and/or added complexity to their operation. Recently, De Ninno et al. [30] showed the discrimination of live, necrotic, and apoptotic lymphoma cells interrogated at multiple frequencies in a continuous flow-through cytometer with coplanar electrodes.

This study seeks to build upon the aforementioned works to develop a microfluidic impedance cytometer capable of Jurkat cell viability discrimination that is optimized for sensitivity and simplicity with the use of widely accessible fabrication techniques and instrumentation. The specific objectives of this study were to: (1) design and evaluate a microfluidic impedance cytometer with parallel vertical sidewall electrodes fabricated using a widely accessible manufacturing technique without the inclusion of constriction channels, particle focusing, or other convoluting or limiting features; (2) optimize device configuration and operation conditions for sensitive viability testing by use of a custom cell buffer and optimum excitation frequency selection; (3) demonstrate reliable discrimination of live and dead cell status using a custom signal processing algorithm; and (4) compare optical imaging of cells to qualitatively validate theoretical basis of dielectric live/dead discrimination.

## 2. Materials and Methods

### 2.1. Simulations of Electrode Geometries

Computational simulations of parallel and coplanar electrode geometries were generated in the AC/DC module in COMSOL Multiphysics (version 5.3, COMSOL Inc., Stockholm, Sweden) by adapting the approach described by Cottet et al. [31] (additional details provided in Appendix A). This method involves the approximation of a single cell as an 11 µm diameter spherical region of the microchannel with electrical properties differing from those of the fluid in the channel and corresponding to the estimated values expected to be exhibited by the cell of interest. Electrode geometry parameters were kept consistent between each model for congruency. For both configurations, the electrode diameters and spacing were 76 µm and 50 µm, respectively. The microchannel height and width were 80 µm and 50 µm, respectively. Synthetic impedance signals consisting of 50 data points longitudinally at 20 discrete distances relative to the electrodes were taken for each geometry.

### 2.2. Microfluidic Device Design and Fabrication

**Figure 1:**
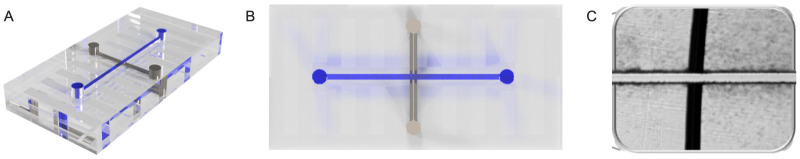
(**A**) Illustration of the microfluidic device with perpendicular fluidic microchannel and metal electrodes along with strain relief structures. (**B**) Simplified two-dimensional (2D) drawing highlighting microchannel-electrodes crossing pattern, and (**C**) microscope image showing the intersection of cylindrical 76 μm diameter Pt electrodes and 50 μm diameter microchannel for fluid perfusion.

A microfluidic device comprised of a single microchannel in a poly(methyl methacrylate), PMMA, chip with a pair of vertical platinum (Pt) side-wall electrodes (Figure 1A) was fabricated using methods adapted from Adams et al. [32]. The microfabrication procedure involved micromilling (KERN MMP 522, Kern Microtechnik GmbH, Murnau, Germany) holes in a 3.2 mm thick PMMA sheet (GoodFellow Corp, Pittsburgh, PA, USA). Pt wires (76 μm, Sigma Aldrich, St. Louis, MO, USA) were threaded into the holes in the PMMA and hot-embossed at 160 °C for 4 min to embed the wire into the polymer. A single 50 μm-wide microchannel was then milled through the PMMA and wire, orthogonally to the wire orientation, to create access for fluid samples to be passed directly between the two cut edges of the wire that comprise sidewall electrodes (Figure 1C).

### 2.3. Cell Culture and Buffer

As a model for human T-lymphocyte cells, Jurkat cells (E6-1, ATCC, Manassas, VA, USA) were used in this study, maintained in 25 cm^2^ flasks with 5 mL of RPMI-1640 culture media (Sigma-Aldrich, St. Louis, MO, USA) containing 10% fetal bovine serum (FBS, Sigma-Aldrich, St. Louis, MO, USA) in an incubator at 37 °C and 5% CO_2_. The dead cell control condition involved incubating cells in a 12-well plate in a solution of RPMI containing 4 mM hydrogen peroxide for 18 h. A custom buffer solution at a pH of 7.4 and approximately 300 mOsm was created for experimentation. All cell impedance measurements were performed in this solution unless otherwise stated.

The pH, osmolarity, and conductivity of relevant fluids—air, deioinized water, the custom buffer, consisting of TRIS, glycine, sucrose and 1% bovine serum albumin—were measured at room temperature prior to impedance testing. The conductivities of buffer solutions were analyzed using an Omega #VDH-7X conductivity meter (Omega, Stamford, CT, USA), and osmolality was measured using a Wescor #5520 osmometer (Wescor Inc., Logan, UT, USA). Frequency sweeps from 20 kHz to 2 MHz were applied to the media, and impedance magnitude and phase data were collected using the impedance analyzer described below.

### 2.4. Flow Cytometry

Flow cytometry was performed for comparison of cell viability in low-conductivity buffers using a FACSCaliber flow cytometer (BD Biosciences, San Jose, CA, USA) using a 488 nm laser. A viability test was performed in which cells were incubated in various buffers—RPMI + FBS, the custom buffer (TRIS + glycine + sucrose + BSA, 1 mM PBS, and 4 mM hydrogen peroxide—for 30 min, 4 h, and 24 h. Cells were labeled with 2 µM Calcein in PBS, washed, and resuspended in 300 μL PBS. Fluorescence data were collected from 30,000 cells per sample and recorded with Cellquest Pro software (BD Biosciences, San Jose, CA, USA). Scatter gating was performed using data from live cell control samples.

### 2.5. Impedance Data Collection and Processing

Fluid flow into devices was provided by programmable NE-500 syringe pumps (New Era Pump Systems, Inc., Wantagh, NY, USA) at a flow rate of 1 μL/h. Concentrations of cells in buffered solutions were fixed at 1 × 10^5^ cells mL^−1^ to minimize the risk of coincidence detection events and to be compatible with video microscopy instrumentation. Electrical signals from the microdevice were acquired using an Agilent E4980A Precision LCR Meter (Agilent Technologies, Irvine, CA, USA) with a supplied voltage of 1 V. The impedance analyzer was self-calibrated using short and open calibrations before the samples were analyzed. For control sample impedance measurements, short measurement scans were performed at 1.0, 1.5, and 2.0 MHz. A custom-programmed National Instruments VI (National Instruments, Austin, TX, USA) was used to collect impedance data. The data was further processed by correlation to the appropriate mathematical function using a custom signal processing algorithm developed in MATLAB (R2019b, The MathWorks, Inc., Natick, MA, USA).

### 2.6. Video Microscopy

Cell viability during impedance cell detection events was confirmed using brightfield and fluorescence video microscopy. Live and dead cell viability control treatments were conducted, wherein Calcein AM was used to assess cell viability. In the microdevice, fluorescent video microscopy acquisition was acquired at a frame rate of 3 frames per second (fps) and an exposure time of 250 ms. Fluorescence microscopy was performed using an inverted Eclipse TS100 Nikon fluorescence microscope(Nikon, Tokyo, Japan), and image stacks were recorded on a CoolSnapFX camera (Photometrics, Tucson, AZ, USA). Image stacks were processed using MetaVue software (Universal Imaging Corporation, West Chester, PA, USA) on a Windows computer.

### 2.7. Electron Microscopy

Electron microscopy was used to correlate the impedance measurements to biophysical properties pertinent to viability discrimination such as membrane morphology and cell size. Cultured cells were collected into a solution with 4% glutaraldehyde in 0.1 M sodium cacodylate buffer. Then, the solution was further diluted in 2% glutaraldehyde in 0.1 M sodium cacodylate buffer, pushed through polycarbonate filter paper (5 μm pore size), and fixed for 4 h. The suspension was then washed four times in 0.1 M cacodylate buffer containing 0.02 M glycine, post-fixed in 2% osmium tetroxide, rinsed in water, en bloc stained in 0.5% uranyl acetate in the dark, rinsed again in water, and dehydrated in ethanol. For transmission electron microscopy (TEM), one half of the sample was infiltrated in 1:1 ethanol:LR White resin solution, infiltrated in pure LR White resin, and embedded in LR White overnight. Ultrathin sections were cut using an Ultracut E ultramicrotome (Reichert-Jung, Austria), mounted on collodion-coated copper grids, stained with Reynolds lead citrate, and examined with a JEOL 100CX transmission electron microscope (JEOL Ltd., Tokyo, Japan) at 80 kV. The second half of the filter paper was used for scanning electron microscopy (SEM). The sample was twice soaked in hexamethyldisilazane (HMDS), air dried, and mounted on SEM stubs. Cell specimens were coated with 25 nm gold palladium using a Hummer II Sputter Coater (Ladd Research, Williston, VT, USA) and examined on a Cambridge S-260 scanning electron microscope (Leica Microsystems, Deerfield, IL, USA).

## 3. Results and Discussion

### 3.1. Simulations of Vertical and Coplanar Electrode Geometries

A device employing a single pair of electrodes was used to minimize the complexity of fabrication, operation, and signal processing. This configuration also offers greater sensitivity than any other configuration implementing more than two electrodes, as the volume of the induced electric field is minimized, thereby maximizing the volume fraction occupied by cells. Additionally, the minimization of the volume of the interrogation region reduces the probability of a coincidence event in which multiple cells are interrogated simultaneously.

**Figure 2:**
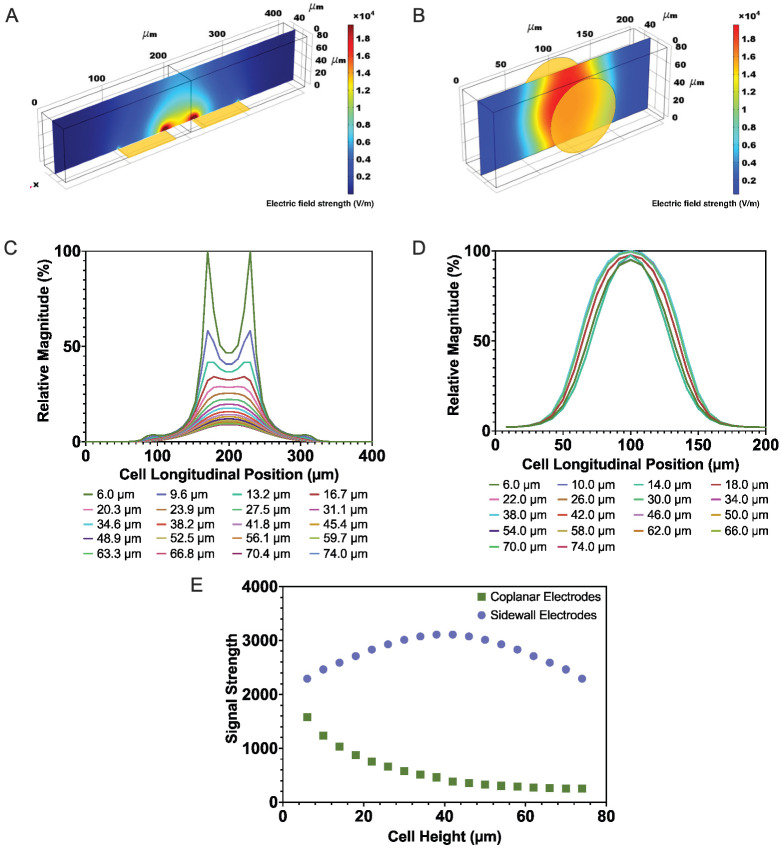
(**A**) Map of the electric field strength through the microfluidic channel centerline created by coplanar electrode geometry, (**B**) map of electric field strength through the microchannel centerline created by parallel electrode geometry, (**C**) relative impedance magnitude variation (%) of insulating sphere passing between coplanar electrodes, (**D**) relative impedance magnitude variation (%) of insulating sphere passing between parallel electrodes, and (**E**) areas under the curves corresponding to each impedance magnitude profile from both electrode geometries. Each plot line corresponds to a pathway through the channel at the longitudinal centerline at various heights ranging from the top to the bottom of the microchannel. Cell heights and Z values refer to positions of the center of the insulating sphere.

Finite element analysis has been proven to be a useful tool for the study of various electrode configurations and microfluidic geometries for impedance cytometry. A COMSOL model of the device reported herein including an 11 μm diameter spherical region with appropriate dielectric properties representing a Jurkat cell was generated to evaluate the efficacy of the Gaussian function for application of the correlation method. A second model with similar dimensions, yet coplanar electrode orientation, was evaluated to compare the vertical positional dependence between the two approaches. Figure 2 shows the distributions of the electric field strengths and relative impedance signals resulting from translation of an insulating sphere through each of these geometries at varying heights (where cell heights and Z values refer to the vertical distance of the center of the cell) from the channel floor.

As illustrated by the electric field maps (Figure 2A,B), the strength of the electric field from the parallel sidewall electrodes is better maintained through the height of the channel. This phenomenon, which is reflected by the larger relative amplitudes of the weakest impedance signal created by a cell passing between the sidewall electrodes compared to the weakest signal created by the coplanar electrodes (Figure 2C,D), demonstrates the improved sensitivity achieved by the vertical parallel electrode configuration.

To compare the vertical positional dependencies of impedance signals generated by the two electrode configurations independent of any curve fitting, the strengths of the impedance magnitude signals were measured in terms of the area under the curves (similar to the root mean square analyses commonly performed on power spectral density data). For congruence, these area values (Figure 2E) were calculated based on the relative impedance magnitude variations and normalized cell longitudinal positions. The difference in these areas between the strongest and weakest signals from each of the parallel and coplanar electrode configurations were 21.5% and 83.7%, respectively. This demonstrates that the relative uniformity in the electric field created by the sidewall electrode geometry leads to more reliable and accurate measurement capability. Additionally, the inconsistency in the shape of the impedance profiles that occur at different heights in the channel between coplanar electrodes are not suitable for signal processing methods that rely on the correlation method; as a result, processing signals obtained by devices using this configuration can be a significant obstacle.

The quality of the fit of the expected impedance signals at various heights to a Gaussian function was also evaluated using goodness of fit (R^2^) values. For the electrode and microchannel geometry used in this study, the R^2^ values for pure magnitude profiles was 0.993 ± 0.004. This suggests that the single pulse Gaussian profile is an appropriate template function for the extraction of cell detection events from raw impedance signals. As a result, this function was used for the signal processing algorithms employed in this study. To evaluate the effect of noise on the quality of the fit, artificial noise was added to the impedance profiles using a periodic function and random number generator in Matlab at an amplitude that corresponded to the noise observed from the measurement system used in this study. The resulting average goodness of fit value with the added noise was 0.821 ± 0.094.

### 3.2. Cell Buffer

To maintain cell viability and achieve impedance data collection, cell media must be optimized in terms of osmolarity, pH, and conductivity. Optimal cell buffer properties include physiological osmolarity (300 mOsm) and physiological pH (7.4). To maximize device sensitivity, it is advantageous to choose a buffer that maximizes the interactions of cells with the electrical energy applied via electrodes. That is, the media should not be so conductive that the energy preferentially bypasses the cells. Phosphate-buffered saline (PBS), sometimes supplemented with sugars such as sucrose or dextrose, is often used as the suspension media for impedance spectroscopy and cytometry [20,33]. To optimize the conditions described above for cell viability and sensitive impedance measurements, a custom low conductivity buffer containing 25 mM Tris, 192 mM Glycine, 83 mM Sucrose, and 1% BSA was developed. As shown in Table 1, relevant properties of various cell buffers were analyzed in the microfluidic device for comparison to the custom buffer and for determination of the optimum conditions for maintaining and measuring cell viability.

As shown, the pH of the diluted (1 mM) PBS matched the pH of 1× PBS, and therefore physiological pH, while the custom buffer was found to have a pH close to, but not exactly matching, that target. The osmolality of the custom buffer closely matches that of 1× PBS, and therefore physiological conditions, while 1 mM PBS showed a significantly lower value.

### 3.3. Flow Cytometry

**Figure 3:**
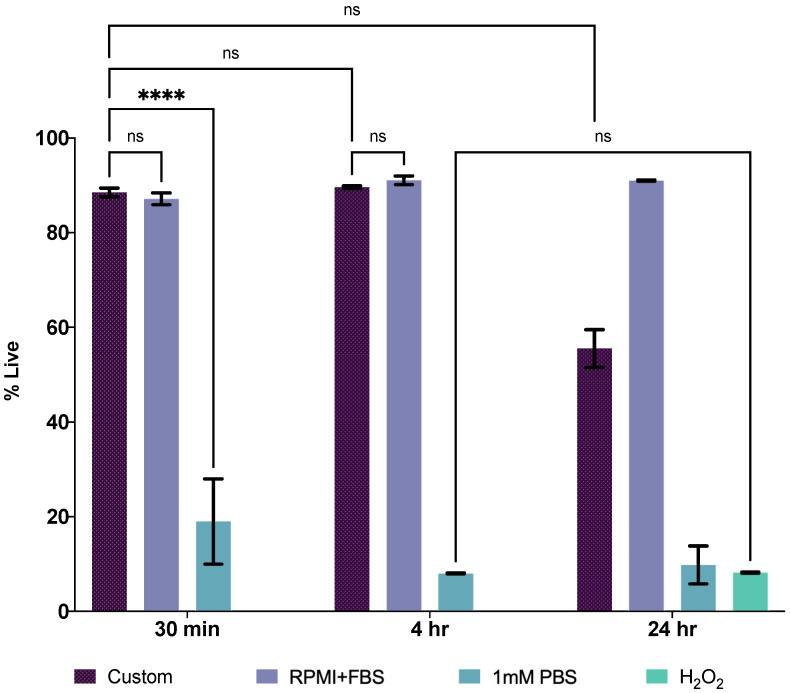
Time course flow cytometry viability analysis of Jurkat cells in RPMI + FBS (live, positive control), TrisGly + Sucrose + BSA, 1 mM PBS, and 4 mM H2O2 (dead, negative control). Flow cytometry data gating was performed on Calcein + cells (30,000 cell events collected; n = 3). Asterisks denote results from statistical comparisons (two-way ANOVA) where **** indicates a *p* value ≤ 0.0001.

Flow cytometry was performed on Calcein-labeled cells suspended in two low-conductivity buffers (1 mM PBS and the custom solution) at three time points (30 min, 4 h, and 24 h) to evaluate the buffers’ effects on cell viability. These buffers were compared to positive (viable) and negative (necrotic) control treatments, RPMI + FBS and H_2_O_2_, respectively. Time points were chosen based on typical duration of experimentation and observation, where 4 h is the longest time any samples remained in the low-conductivity buffer. Figure 3 shows the fraction of viable cells in each sample at these time points.

Statistical comparisons were performed using two-way ANOVA comparing % live values for each sample group across time. Cells maintained in 1 mM PBS did not maintain viability (8.28 ± 0.41%) for any duration relevant to the required testing for this study and demonstrated no statistical difference from the necrotic treatment after 24 h. Conversely, there was no significant difference between cell viability in the custom Tris-Glycine + Sucrose + BSA buffer (88.50 ± 0.92% at 30 min and 89.66 ± 0.28% at 4 h) and the positive control (87.15 ± 1.24% at 30 min and 91.06 ± 0.91% at 4 h) during the experimentation time window. Based on the data shown in Table 1 and Figure 3, the custom Tris-Glycine + Sucrose + BSA buffer was selected for subsequent cell-based impedance measurements as this solution exhibited the lowest measured conductivity while also closely matching physiological conditions of osmolarity and pH.

### 3.4. Identification of Optimum Frequency

**Figure 4:**
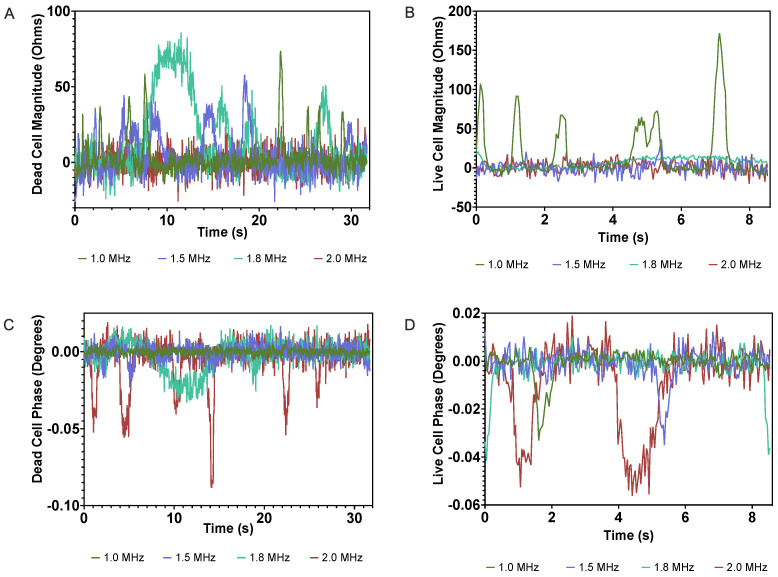
Relative impedance magnitude changes of (**A**) dead and (**B**) live cells at 1.0, 1.5, and 2 MHz interrogation frequencies. Relative impedance phase changes of (**C**) dead and (**D**) live cells.

In a previous report of a flow-through application of microfluidic impedance determination of T lymphocyte cell viability [30], samples were interrogated at 0.5 and 10 MHz. While multifrequency analyses using low and high frequencies can simultaneously uncover useful information about more subtle cellular properties, the use of instrumentation capable of such multifrequency analyses may be inaccessible financially or otherwise for some prospective users. As a result, one goal of this study was to present an alternative approach by identifying a single frequency at which the impedance signatures cells could be easily detected and viability status accurately discriminated on a continuous basis. Impedance measurements for live and dead cell control samples at several frequencies were collected independently and analyzed. Frequencies at the lower end of the intermediate frequency range (described above) up to the highest achievable frequency by the Agilent LCR Meter (2 MHz) were chosen in an attempt to capture both the resistive and capacitive behaviors of interrogated cells. Impedance magnitude and phase signals were collected for live and dead cell control samples at frequencies of 1.0, 1.5, and 2.0 MHz, as shown in Figure 4.

When compared with video microscopy, live single-cell events were observed to correlate with observable impedance phase signal changes, while dead single cell events displayed a change in the impedance magnitude at all frequencies tested. Importantly, at 2 MHz, live and dead cells displayed those expected dielectric responses without displaying the converse behaviors. That is, at this frequency, interrogation of live single cells created changes in the measured impedance phase without any appreciable change in magnitude while dead cells induced impedance magnitude changes without any significant phase change. Compared to previously reported devices for viability testing of T lymphocyte cells, the selection and application of an electric field at an optimum frequency allows for the interrogation of cells at a single frequency, thereby removing the need for more complex equipment capable of multifrequency analyses.

**Figure 5:**
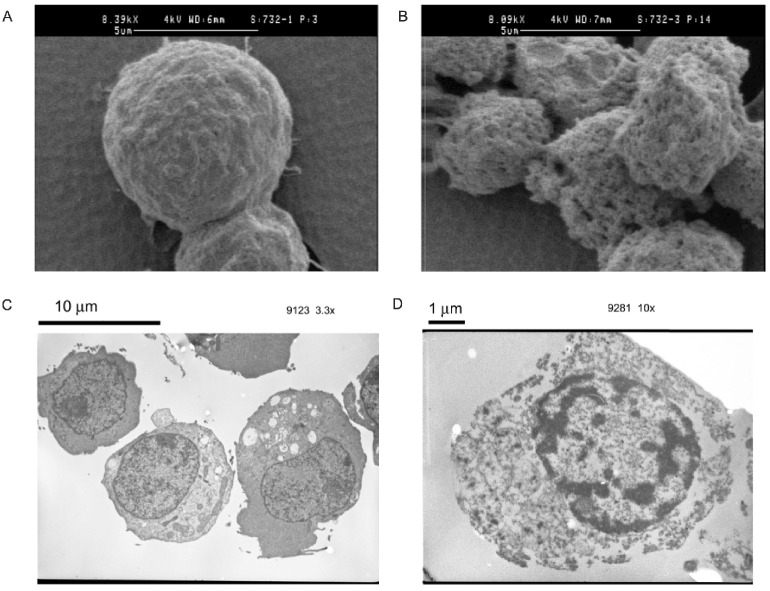
SEM images of Jurkat cells including a (**A**) Live cell and a (**B**) Dead (4 mM H_2_O_2_-treated) cell (scale bar: 5 μm). TEM images of Jurkat cells (**C**) Live cell (scale bar: 10 μm), (**D**) Dead (4 mM H_2_O_2_-treated) cell (scale bar: 1 μm).

The phenomena presented above can be explained by relating the frequency-dependence of the mechanism of cells’ interactions with electrical current to the biophysical changes in cell structures that occur upon cell death. Wang et al. [34] observed the changes in HL-60 cell membranes during apoptosis using SEM and measured a corresponding change in membrane capacitance. The changes that occurred during apoptosis were found to include an increase in the smoothness of the membrane surface associated with loss of membrane features (e.g., microvilli). This decrease in morphological complexity corresponded to a decrease in measured capacitance. Additionally, necrotic HL-60 cells were found to have a different crossover frequency and a larger surface area from live cells, which supports the differences in dielectric behaviors observed. Similarly, as shown in Figure 5, SEM images for live Jurkat cells (Figure 5A) present a rounded morphology with characteristic folds in their membranes, whereas dead cells (Figure 5B) exhibit more nonuniform morphologies with perforated outer membranes. TEM images further highlight the disrupted cell membranes of the dead cells (Figure 5D) whereas preservation of the intact cell membranes can be seen in the live cells (Figure 5C).

In low MHz ranges, alternating currents can induce polarization of membrane ions in live cells, leading to membrane capacitance, which is proportional to the amplitude of changes in impedance phase. Because the membrane is disrupted upon cell death, the ion polarization events that lead to capacitance do not occur. Instead, the electric current interacts predominantly with the cell based on its resistivity, which is likely to increase due to the increase in surface area and loss of intracellular ions. As a result, combining the consideration of the effects of the frequency of the applied electric current on the cell being analyzed and the effects of cell death on cell biophysical characteristics leads to the conclusion that alive and dead cells of the same type can exhibit sufficiently different impedimetric behaviors at a single frequency to discriminate between the two populations. Therefore, at an optimum frequency (in this case, 2 MHz), live cells can be discriminated simply by observing relative changes in the impedance phase with no change in magnitude, while dead cells show relative changes in impedance magnitude with no change in phase.

As illustrated above, raw output signals often have baseline drift and high levels of noise that can make extracting useful information challenging. As a result, although the data samples shown were chosen for illustration based on their clarity, quantitative identification of cell detection events is often difficult to achieve reliably. A few methods have been demonstrated previously for digital impedance signal processing, including Savitzky–Golay smoothing [35] and the correlation method [36]. Savitzky–Golay smoothing, a widely used alternative to moving average filtering for noise reduction in some fields (e.g., analytical chemistry), applies a low pass polynomial filter by mathematical convolution and is dependent on an input order and frame length.

**Figure 6:**
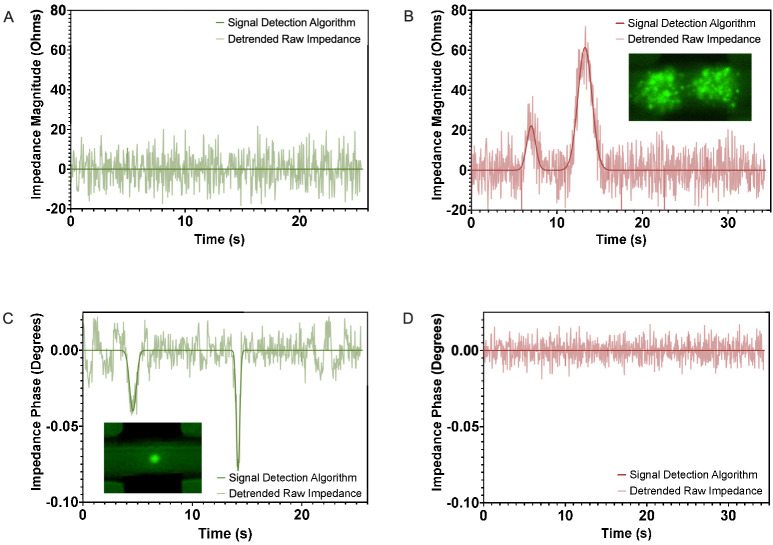
Relative changes in (**A**) impedance magnitude of live cells, (**B**) impedance magnitude of dead cells, (**C**) impedance phase of live cells, and (**D**) impedance phase of dead cells. Raw and filtered data are shown for each dataset. Stills from fluorescence video microscopy corresponding to dead and live cell detection events are shown in the corresponding windows.

To illustrate the limitations of commonly used smoothing techniques, the results of moving average and Savitzky–Golay processing methods were applied to characteristic signals collected during this study. Moving average filters were applied with window sizes of 5 and 50 to explore the effects of small and large window sizes, respectively, while 3 combinations of orders and frame lengths for Savitzky–Golay filtering were applied to illustrate their effects. As shown in Figure 6, moving average filters that incorporate too few data points do not adequately smooth noisy data. Conversely, as the window size is increased, the amplitudes of signal peaks are dampened. This leads to reduced sensitivity and an increased chance of missed cell detection events.

In Savitzky–Golay smoothing, there exists a similar compromise between noise reduction and preservation of peak amplitudes. Higher order filters do tend to maintain peak values but at the expense of noise reduction. Longer, lower-order filters, conversely, tend to more effectively reduce signal noise but dampen peak amplitude values. Identification of optimum order and frame length settings for noise reduction and peak preservation can be difficult or, in some cases, unachievable [25].

As a result of these limitations, mathematical correlation to a Gaussian template function was implemented using a custom Matlab algorithm. This algorithm segments data based on cell driving velocity and the volume of the device’s interrogation region, fits magnitude and phase data segment to the template function, and compares the goodness of fit values to a threshold determined by analysis of control samples. Extracted features are kept while segments identified as noise are passed through a polynomial filter to remove the baseline drift and noise.

### 3.5. Live and Dead Control Sample Interrogation at 2 MHz

**Figure 7:**
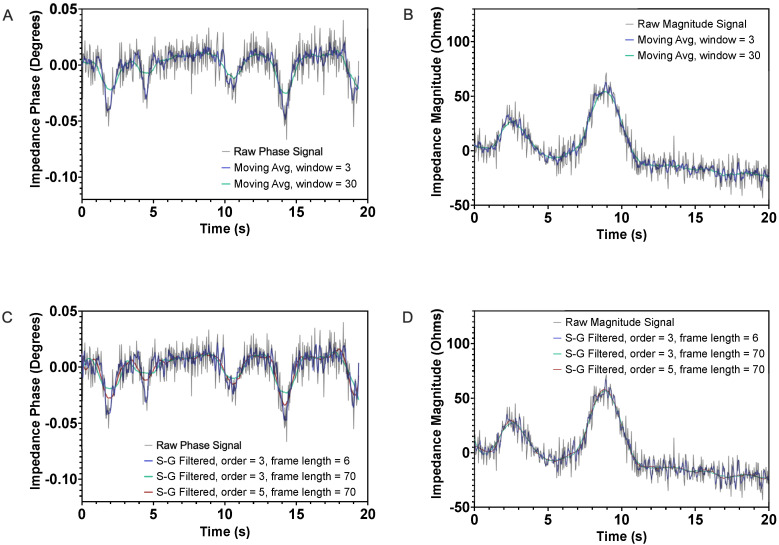
Signal detection algorithm output plotted against detrended noisy impedance output profiles corresponding to (**A**) impedance magnitude of live cells, (**B**) impedance magnitude of dead cells, (**C**) impedance phase of live cells, and (**D**) impedance phase of dead cells.

Once the frequency appropriate for cell status discrimination was determined, live and dead cell control samples were tested at an excitation frequency of 2 MHz, and observations were validated by comparison to fluorescence microscopy. Figure 7 shows the processed, detrended data corresponding to raw data shown in Figure 4, plotted against the complete output of the signal detection algorithm.

At 2 MHz, cell viability status can easily be discriminated by identifying detection events in impedance magnitude and phase profiles. Live cells clearly display impedance phase changes with no discernible impedance magnitude change, whereas dead cells display impedance magnitude changes with no evident changes in impedance phase. Additionally, the signal detection algorithm is able to effectively process raw data to eliminate baseline drift and identify cell detection events without reducing the amplitude of signal peaks, despite significant noise. Based on the combination of the instrumentation and the microfluidic chip geometry utilized, an estimated maximum throughput for the detection of Jurkat cell viability is 1800 cells per minute. While this rate is limited by the sampling rate of the hardware used, it demonstrates the possibility of collecting population-level statistics using relatively low-cost electrical analysis hardware.

**Figure 8:**
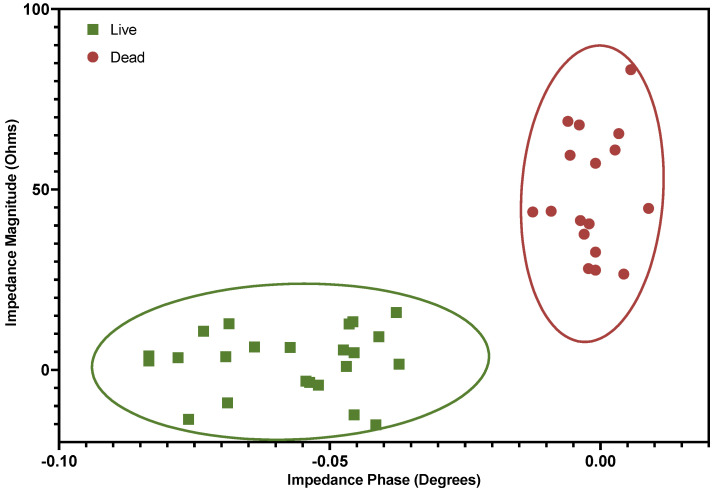
Representative showing that Jurkat cell viability status can be discriminated using the designed microchip device with impedance measurements at 2 MHz by plotting impedance components magnitude and phase against one another. Ellipses indicate 95% confidence inervals.

Discrimination of cell viability states using the approach presented in this study can be more clearly demonstrated by plotting impedance magnitude and phase values (for live and dead cells) against one another using a scatter plot. To accomplish this, the signal processing algorithm was amended to record the peak magnitude of every positive signal detection event from collections of live and dead cells. That is, the algorithm identified the signal peak corresponding to the impedance component that adequately displays Gaussian behavior, relative to the prescribed threshold for detection, and records the amplitude and index of that peak. If the converse impedance component does not adequately display Gaussian behavior as expected, that signal is averaged over a window of data points one-tenth of the data segment in length. Figure 8 shows the measured impedance magnitude and phase outputs resulting from signal processing plotted against one another.

This plot also contained 95% confidence ellipses for the sampled data collected from live and dead cell samples. Calculated in Matlab, the widths and angles of the major and minor axes of the ellipses were determined by the maximum and minimum eigenvalues of the covariance matrices of phase and magnitude values. As shown above, Jurkat cell viability can be determined for at least 95% of interrogated cells using this device and signal detection algorithm.

## 4. Conclusions

This paper presents a microfluidic impedance cytometer optimized for discrimination of the viability status of Jurkat cells using a device optimized for sensitivity and accuracy without the use of complex elements or expensive equipment. This tool achieves effective discrimination of live and dead cells using a vertical sidewall electrode geometry that generates a vertically uniform electric field. This parallel sidewall geometry mitigates the problems associated with positional dependence and current leakage, which are more prominent in coplanar electrode geometries. The electrodes were fabricated using an accessible, CAD-driven negative manufacturing process, demonstrating its potential for more widespread use. Furthermore, because the fundamental mechanisms and operation of this device do not require complex particle focusing or trapping equipment, this device provides a simple platform for single cell biophysical property analysis. Finally, the inclusion of a custom buffer solution highlights a potential mitigating action that can be used to overcome the challenges of sensitivity and current leakage associated with impedance cytometry platforms.

The device design and implementation methods used herein have limitations that should be considered in subsequent applications. First, the diameter of the microelectrodes must be equal to or larger than the diameter of the microchannel to prevent cells from passing undetected beyond the electrode boundaries. Additionally, the throughput of the data collection reported was restricted well below those achievable by commercially available optical flow cytometry instruments due to the limitations of the impedance analysis instrumentation and congruent fluorescent video microscopy. However, the use of more advanced electronics can be implemented to achieve throughputs comparable to optical flow cytometry. Finally, determination of the optimum frequency for viability testing is a cell type-specific methodology that would need to be performed on an *ad hoc* basis. Overall, however, the general approach taken by this study is easily amenable for other applications of microfluidic impedance cytometry for single cell analysis. Simple modifications to the applied frequency, electrode size, cell suspension buffer, and signal processing algorithm can allow for interrogation of many cell types and extraction of a number of important biophysical properties on a single-cell basis.

## Figures and Tables

**Table 1:** Properties of cell buffers important to cell viability maintenance and sensitive impedance detection.

Cell Buffer Solution	Conductivity	Osmolality	pH
	(μS/cm)	(mM/kg)	
dI Water	2 ± 40	0	∼6.4
25 mM Tris, 192 mM Glycine	350 ± 40	180	8.3
25 mM Tris, 192 mM Glycine + 83 mM Sucrose + 1% BSA	1200 ± 40	292	7.6
0.1× PBS	1200 ± 40	56	7.4
1× PBS	9300 ± 40	300	7.4

**Figure A1:**
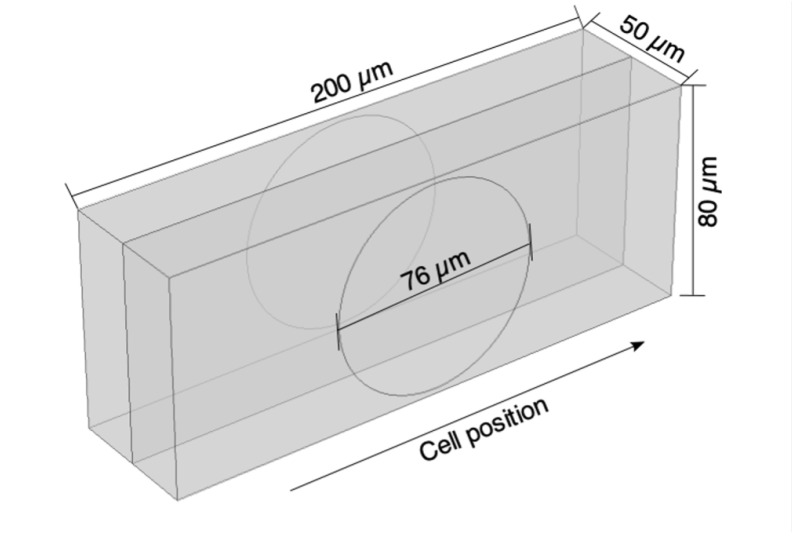
Schematic diagram of the model geometry representing the device design used in this study.

**Figure A2:**
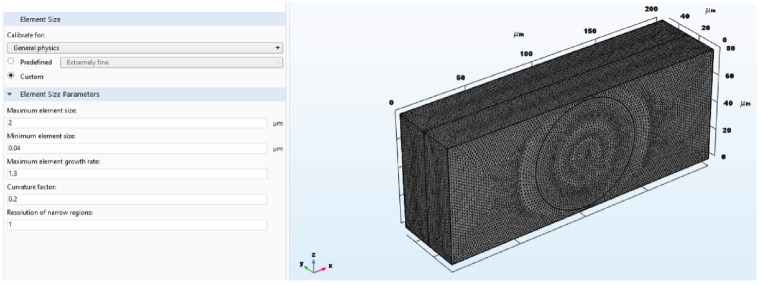
Meshing parameters and completed mesh of model geometry.

## Data Availability

The datasets generated during and/or analyzed during the current study are available from the corresponding author on reasonable request.

## Abbreviations

The following abbreviations are used in this manuscript:

FACS	Fluorescence-activated cell sorting
AC	Alternating current
DC	Direct current
PMMA	Poly(methyl methacrylate)
FBS	Fetal bovine serum
dI	Deionized
TRIS	Tris(hydroxymethyl)aminomethane
BSA	Bovine serum albumin
PBS	Phosphate-buffered saline
TEM	Transmission electron microscopy
HMDS	Hexamethyldisilazine
SEM	Scanning electron microscopy
CAD	Computer-aided design

## Appendix A

### Appendix Simulation of Device Design

Computational simulations were created to model the impedance signal generated by a cell passing through the interrogation region of the device presented in this study. The method is described by Cottet et al. [31] and involves defining a region of the model geometry as having dielectric properties corresponding to the cell of interest distinct from those of the cell-carrying buffer. The method was adapted herein.

A 200 μm length of the microchannel was selected to encompass the entire electric field in the system. Although the actual height of the microchannel may vary due to limits to the resolution of micromilling instrumentation and the extents of surface modification resulting from hot embossing, the model geometry was specified to have a height of 80 μm to encompass the full diameter of the electrodes, thereby revealing the extent of potential positional variance in measured impedance signals. The circles on the periphery of the geometry represent the area of the interface between the platinum electrodes and the fluid microchannel. One of these represent the excitation electrode and is defined as an electrical terminal with an applied voltage of 1 V, while the other represents the sensing electrode and is defined as a ground boundary.

The input specifications used to define the material properties are described below.

**Table A1:** Dielectric material properties used in the computational model of the reported device.

Property	Unit	Value	Basis
Solution Conductivity	S/m	0.12	Conductivity of custom TrisGly + Sucrose + BSA buffer used in this study
Solution Relative Permittivity	–	80	Relative permittivity based on properties of PBS and custom buffer [37]
Particle Conductivity	S/m	10^−12^	Median value of commonly reported membrane conductivities of biological cells
Particle Relative Conductivity	–	6	Median value of commonly reported membrane conductivities of biological cells

The radius of the cell is defined as r0, and the position of its center is defined using Cartesian coordinates (x0, y0, z0). The properties of the material in the model are then defined using a conditional expression that applies the properties of the “particle” (or cell) within the radius of the cell and the properties of the “solution” outside of that region. The movement of the cell is achieved by varying the position of its center by using an auxiliary parametric sweep.

A free tetrahedral mesh element shape with the following size settings was used throughout the full extent of the geometry. Standard adaptive meshing was used, but no boundary layer or other distributive modifications were made. The mesh element size settings were established as follows:

COMSOL’s AC/DC module calculates the electrical properties as lumped parameters. When a voltage is applied in the Electrostatics physics package, an admittance matrix, Y, is determined. Specifically, as only one voltage is applied, the system admittance is represented as the first element in Y, Y11. Therefore, by definition, the system impedance can be calculated by defining a new variable as Z11 = 1/Y11 and by assigning a global variable probe. Because impedance data is measured as the cell is outside of the induced electric field, the contribution of the buffer impedance to the synthetic data stream can be removed.

Experimental data was collected using an Agilent E4980A LCR Meter controlled by LabView. Output impedance magnitude and phase data was collected and imported into Matlab. An algorithm was developed to process control sample data by a three-step process. First, a segment of data is taken, whose length is based on the velocity of the cells in the microchannel and the length of the detection region. The data segment is then fitted against a mathematical single pulse Gaussian function, of the form

$$y_{pred}=a*exp{\left(\left(t-b\right)/c\right)}^{2}$$

where *t* is the time in seconds; *a*, *b*, and *c* are the fitting parameters. The quality of the fits of data segments corresponding to cell detection events (as confirmed by video microscopy) were recorded. Data that does not correspond to a cell event is passed by the algorithm as noise and is detrended to remove the baseline drift. The data segments are then recombined and expressed together. No data smoothing is applied to avoid artificially suppressing important trends.
